# Tris­(4,4′-di-*tert*-butyl-2,2′-bi­pyridine)(*trans*-4-*tert*-butyl­cyclo­hexa­nolato)­deca-μ-oxido-hepta­oxido­hepta­vanadium aceto­nitrile monosolvate including another unknown solvent mol­ecule

**DOI:** 10.1107/S2414314620004496

**Published:** 2020-04-07

**Authors:** Shintaro Kodama, Shota Kondo, Akihiro Nomoto, Akiya Ogawa

**Affiliations:** a Department of Applied Chemistry, Graduate School of Engineering, Osaka Prefecture University, 1-1 Gakuen-cho, Nakaku, Sakai, Osaka 599-8531, Japan; Okayama University, Japan

**Keywords:** crystal structure, vanadium oxide cluster, bi­pyridine, alcohol

## Abstract

The title hepta­nuclear alkoxido(oxido)vanadium(V) oxide cluster complex, [V_7_(C_10_H_19_O)O_17_(C_18_H_24_N_2_)_3_]·CH_3_CN, was obtained by the reaction of [V_8_O_20_(C_18_H_24_N_2_)_4_] with 4-*tert*-butyl­cyclo­hexa­nol (mixture of *cis* and *trans*) in a mixed CHCl_3_/CH_3_CN solvent. The complex has a V_7_O_18_N_6_ core with approximately *C*_s_ symmetry, which is composed of two VO_4_ tetra­hedra, two VO_6_ octa­hedra and three VO_4_N_2_ octa­hedra. In the crystal, these complexes are linked together by weak inter­molecular C—H⋯O hydrogen bonds between the 4,4′-di-*tert*-butyl-2,2′-bi­pyridine ligand and the V_7_O_18_N_6_ core, forming a one-dimensional network along the *c*-axis direction. Besides the complex, the asymmetric unit contains one CH_3_CN solvent mol­ecule. The contribution of other disordered solvent mol­ecules to the scattering was removed using the SQUEEZE option in *PLATON* [Spek (2015 ▸). *Acta Cryst*. C**71**, 9–18]. The unknown solvent mol­ecules are not considered in the chemical formula and other crystal data.

## Abstract 2

The title hepta­nuclear alkoxido(oxido)vanadium(V) oxide cluster complex, [V_7_(C_10_H_19_O)O_17_(C_18_H_24_N_2_)_3_]·CH_3_CN, has a V_7_O_18_N_6_ core with approximately *C*_s_ symmetry, which is composed of two VO_4_ tetra­hedra, two VO_6_ octa­hedra and three VO_4_N_2_ octa­hedra.

## Graphical abstract

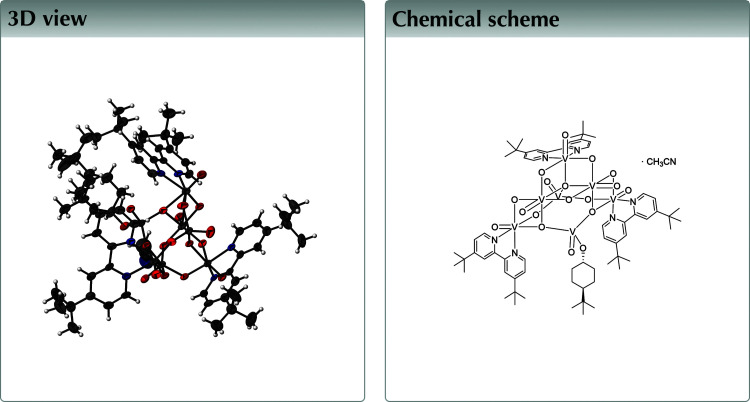

## Structure description

In contrast to anionic vanadium oxide clusters (POVs) (Hayashi, 2011 ▸), isolated non-ionic vanadium oxide clusters are still limited in number, although they have the potential to exhibit chemical properties distinct from those of POVs. In fact, non-ionic vanadium oxide clusters have recently attracted much attention as a catalyst for oxidation of hydro­carbons in a gas-phase study (Dong *et al.*, 2008 ▸). Non-ionic vanadium oxide clusters are typically isolated as organic ligand-functionalized POVs that are often synthesized directly from monomeric oxidovanadium species (Gao *et al.*, 2014 ▸). However, most of them have low solubility in common organic solvents, and therefore their chemical properties and structural transformations in solution still remain largely unexplored.

**Figure 1:**
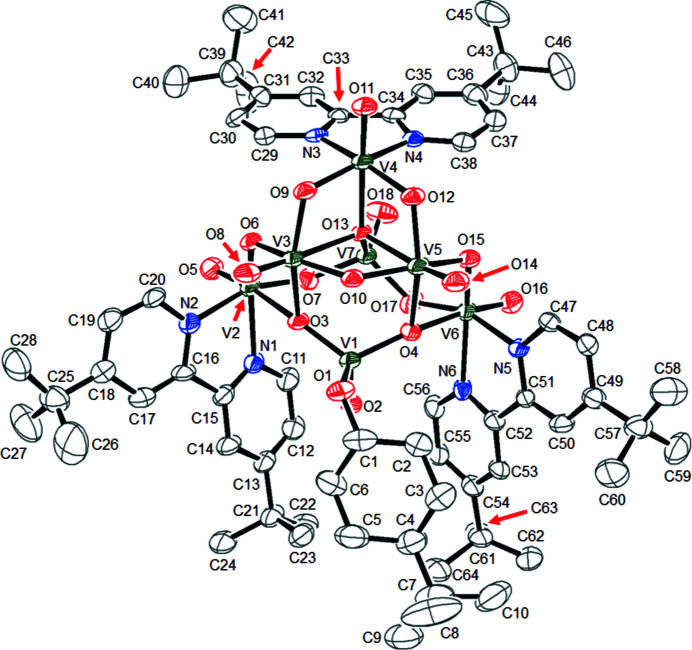
The mol­ecular structure of the title compound, showing the atom-labelling scheme. Displacement ellipsoids are drawn at the 50% probability level. The solvent CH_3_CN mol­ecule and H atoms have been omitted for clarity.

**Figure 2:**
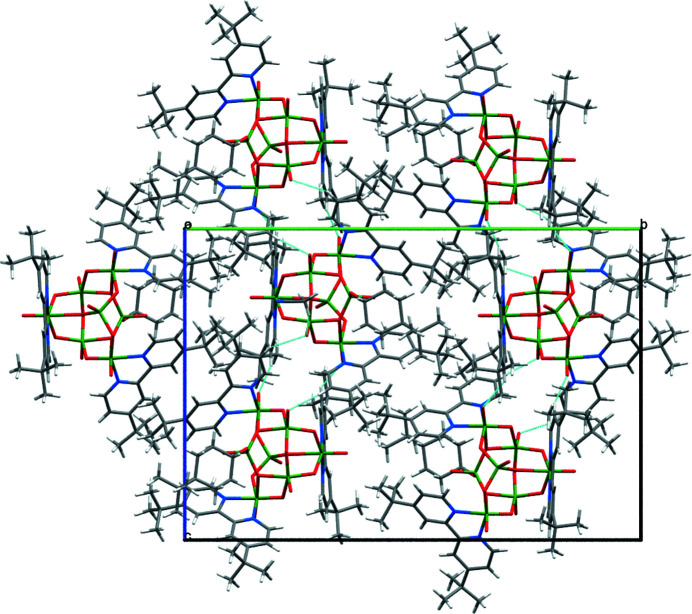
A packing diagram of the title complex viewed along the *a* axis. The C—H⋯O hydrogen bonds are represented by the light blue lines.

Structural transformations of [V_8_O_20_(4,4′-^*t*^Bubpy)_4_] (4,4′-^*t*^Bubpy = 4,4′-di-*tert*-butyl-2,2′-bi­pyridine) in alcoholic solution have been reported (Kodama *et al.*, 2014 ▸, 2016 ▸; Inoue *et al.*, 2018 ▸), where the 2-meth­oxy­ethoxido analogue of the title complex was crystallized from 2-meth­oxy­ethanol solution of [V_8_O_20_(4,4′-^*t*^Bubpy)_4_]. In the present study, single crystals of the title complex were successfully obtained by dissolving [V_8_O_20_(4,4′-^*t*^Bubpy)_4_] and an excess amount of 4-*tert*-butyl­cyclo­hexa­nol (solid at ambient temperature) in a mixed CHCl_3_/CH_3_CN solvent, followed by slow diffusion of diethyl ether into the resulting solution. The mol­ecular structure of the title complex is presented in Fig. 1 ▸. The V_7_O_18_N_6_ core with approximately *C*_s_ symmetry is composed of two VO_4_ tetra­hedra, two VO_6_ octa­hedra, and three VO_4_N_2_ octa­hedra. The V–O_terminal_ distances and the V–O_bridged_ distances are within the range of these bonds found in vanadium oxide clusters, although the V3—O13 [2.476 (3) Å] and V5—O13 [2.527 (3) Å] distances are relatively long (Kodama *et al.*, 2016 ▸; Schindler *et al.*, 2000 ▸). The crystal packing of the title complex is shown in Fig. 2 ▸. Weak inter­molecular C—H⋯O hydrogen bonds were observed between the 4,4′-^*t*^Bubpy ligand and the V_7_O_18_N_6_ core (Table 1 ▸), leading to a one-dimensional network along the *c*-axis direction.

## Synthesis and crystallization

[V_8_O_20_(4,4′-^*t*^Bubpy)_4_]·CH_2_Cl_2_ was prepared according to the procedure reported previously (Kodama *et al.*, 2016 ▸). [V_8_O_20_(4,4′-^*t*^Bubpy)_4_]·CH_2_Cl_2_ (2.8 mg, 0.0015 mmol) and 4-*tert*-butyl­cyclo­hexa­nol (*cis*:*trans* ratio = 33:67) (23.4 mg, 0.15 mmol) were dissolved in a mixed solvent of CHCl_3_ (0.1 mL) and CH_3_CN (0.05 mL) at ambient temperature. Et_2_O was diffused into the resulting solution to give yellow crystals.

## Refinement

Crystal data, data collection and structure refinement details are summarized in Table 2 ▸. Solvent mol­ecules except the refined CH_3_CN mol­ecule were too highly disordered to refine the structure, and so they were treated with SQUEEZE in *PLATON* (Spek, 2015 ▸). This program indicated the total potential solvent-accessible void volume of 2079 Å^3^ per unit cell and 492 electrons/cell within the void.

## Supplementary Material

Crystal structure: contains datablock(s) global, I. DOI: 10.1107/S2414314620004496/is4043sup1.cif

Structure factors: contains datablock(s) I. DOI: 10.1107/S2414314620004496/is4043Isup2.hkl

CCDC reference: 1994012

Additional supporting information:  crystallographic information; 3D view; checkCIF report

## Figures and Tables

**Table 1:** Hydrogen-bond geometry (Å, °)

*D*—H⋯*A*	*D*—H	H⋯*A*	*D*⋯*A*	*D*—H⋯*A*
C19—H19⋯O14^i^	0.95	2.55	3.417 (7)	152
C30—H30⋯O16^i^	0.95	2.38	3.122 (6)	135
C37—H37⋯O5^i^	0.95	2.43	3.264 (7)	146
C40—H40*B*⋯O16^i^	0.98	2.58	3.466 (8)	150
C48—H48⋯O8^i^	0.95	2.32	3.216 (6)	157

Symmetry code: (i) 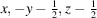 .

**Table 2:** Experimental details

Crystal data	
Chemical formula	[V_7_(C_10_H_19_O)O_17_(C_18_H_24_N_2_)_3_]·C_2_H_3_N
*M*_r_	1630.10
Crystal system, space group	Monoclinic, *P*2_1_/*c*
Temperature (K)	123
*a*, *b*, *c* (Å)	14.6285 (4), 29.9816 (7), 20.5191 (5)
β (°)	94.548 (7)
*V* (Å^3^)	8971.1 (4)
*Z*	4
Radiation type	Mo *K*α
μ (mm^−1^)	0.76
Crystal size (mm)	0.14 × 0.08 × 0.03

Data collection	
Diffractometer	Rigaku R-AXIS RAPID
Absorption correction	Multi-scan (*ABSCOR*; Higashi, 1995 ▸)
*T*_min_, *T*_max_	0.386, 0.978
No. of measured, independent and observed [*F* ^2^ > 2.0σ(*F* ^2^)] reflections	122082, 16426, 12218
*R*_int_	0.113
(sin θ/λ)_max_ (Å^−1^)	0.602

Refinement	
*R*[*F* ^2^ > 2σ(*F* ^2^)], *wR*(*F* ^2^), *S*	0.074, 0.202, 1.02
No. of reflections	16426
No. of parameters	905
H-atom treatment	H-atom parameters constrained
Δρ_max_, Δρ_min_ (e Å^−3^)	1.26, −0.61

Computer programs: *RAPID-AUTO* (Rigaku, 2006 ▸), *SHELXT* (Sheldrick, 2015*a* ▸), *SHELXL2018/3* (Sheldrick, 2015*b* ▸) and *CrystalStructure* (Rigaku, 2018 ▸).

## full crystallographic data

### Crystal data

**Table:**

[V_7_(C_10_H_19_O)O_17_(C_18_H_24_N_2_)_3_]·C_2_H_3_N	*F*(000) = 3376.00
*M**_r_* = 1630.10	*D*_x_ = 1.207 Mg m^−^^3^
Monoclinic, *P*2_1_/*c*	Mo *K*α radiation, λ = 0.71075 Å
*a* = 14.6285 (4) Å	Cell parameters from 76498 reflections
*b* = 29.9816 (7) Å	θ = 1.8–25.4°
*c* = 20.5191 (5) Å	µ = 0.76 mm^−^^1^
β = 94.548 (7)°	*T* = 123 K
*V* = 8971.1 (4) Å^3^	Prism, yellow
*Z* = 4	0.14 × 0.08 × 0.03 mm

### Data collection

**Table:**

Rigaku R-AXIS RAPID diffractometer	12218 reflections with *F*^2^ > 2.0σ(*F*^2^)
Detector resolution: 10.000 pixels mm^-1^	*R*_int_ = 0.113
ω scans	θ_max_ = 25.4°, θ_min_ = 2.3°
Absorption correction: multi-scan (ABSCOR; Higashi, 1995)	*h* = −17→17
*T*_min_ = 0.386, *T*_max_ = 0.978	*k* = −36→36
122082 measured reflections	*l* = −24→24
16426 independent reflections	

### Refinement

**Table:**

Refinement on *F*^2^	Secondary atom site location: difference Fourier map
*R*[*F*^2^ > 2σ(*F*^2^)] = 0.074	Hydrogen site location: inferred from neighbouring sites
*wR*(*F*^2^) = 0.202	H-atom parameters constrained
*S* = 1.01	*w* = 1/[σ^2^(*F*_o_^2^) + (0.088*P*)^2^ + 32.1684*P*] where *P* = (*F*_o_^2^ + 2*F*_c_^2^)/3
16426 reflections	(Δ/σ)_max_ = 0.001
905 parameters	Δρ_max_ = 1.26 e Å^−^^3^
0 restraints	Δρ_min_ = −0.61 e Å^−^^3^
Primary atom site location: structure-invariant direct methods	

### Special details

**Table:**

Geometry. All esds (except the esd in the dihedral angle between two l.s. planes) are estimated using the full covariance matrix. The cell esds are taken into account individually in the estimation of esds in distances, angles and torsion angles; correlations between esds in cell parameters are only used when they are defined by crystal symmetry. An approximate (isotropic) treatment of cell esds is used for estimating esds involving l.s. planes.
Refinement. Refinement was performed using all reflections. The weighted R-factor (wR) and goodness of fit (S) are based on F^2^. R-factor (gt) are based on F. The threshold expression of F^2^ > 2.0 sigma(F^2^) is used only for calculating R-factor (gt).

### Fractional atomic coordinates and isotropic or equivalent isotropic displacement parameters (Å^2^)

**Table:**

	*x*	*y*	*z*	*U*_iso_*/*U*_eq_	
V1	0.65605 (6)	0.63659 (3)	0.78316 (4)	0.0301 (2)	
V2	0.48172 (7)	0.66199 (3)	0.91643 (4)	0.0343 (2)	
V3	0.62439 (6)	0.72445 (3)	0.86078 (4)	0.0311 (2)	
V4	0.52186 (6)	0.80068 (3)	0.77869 (4)	0.0297 (2)	
V5	0.62617 (6)	0.72668 (3)	0.70249 (4)	0.0321 (2)	
V6	0.49956 (7)	0.65816 (3)	0.63388 (4)	0.0333 (2)	
V7	0.41093 (6)	0.68666 (3)	0.76650 (4)	0.0292 (2)	
O1	0.7780 (3)	0.63573 (12)	0.79388 (19)	0.0422 (9)	
O2	0.6242 (3)	0.58546 (11)	0.77893 (16)	0.0348 (8)	
O3	0.6049 (2)	0.65981 (10)	0.85106 (16)	0.0310 (8)	
O4	0.6167 (3)	0.66100 (10)	0.70710 (16)	0.0326 (8)	
O5	0.4081 (3)	0.66153 (12)	0.97006 (18)	0.0416 (9)	
O6	0.5234 (3)	0.71602 (10)	0.91640 (16)	0.0339 (8)	
O7	0.4059 (3)	0.65768 (11)	0.84127 (16)	0.0349 (8)	
O8	0.7080 (3)	0.72585 (11)	0.91539 (19)	0.0414 (9)	
O9	0.5963 (2)	0.78378 (10)	0.84878 (16)	0.0314 (8)	
O10	0.6831 (2)	0.72266 (10)	0.78501 (16)	0.0296 (7)	
O11	0.5317 (3)	0.85395 (11)	0.77907 (17)	0.0358 (8)	
O12	0.5908 (2)	0.78455 (10)	0.71373 (17)	0.0318 (8)	
O13	0.4949 (2)	0.72681 (10)	0.77549 (15)	0.0295 (7)	
O14	0.7090 (3)	0.73244 (11)	0.65780 (18)	0.0402 (9)	
O15	0.5251 (3)	0.71509 (10)	0.63654 (16)	0.0342 (8)	
O16	0.4226 (3)	0.65306 (12)	0.57346 (18)	0.0427 (9)	
O17	0.4320 (3)	0.64820 (12)	0.70402 (18)	0.0428 (9)	
O18	0.3134 (3)	0.71041 (13)	0.7485 (2)	0.0543 (11)	
N1	0.5072 (3)	0.59125 (13)	0.9082 (2)	0.0349 (10)	
N2	0.5886 (3)	0.64562 (14)	0.9907 (2)	0.0372 (10)	
N3	0.4088 (3)	0.80057 (12)	0.8387 (2)	0.0294 (9)	
N4	0.4003 (3)	0.80220 (12)	0.7135 (2)	0.0296 (9)	
N5	0.6079 (3)	0.65007 (12)	0.5689 (2)	0.0323 (10)	
N6	0.5363 (3)	0.58772 (13)	0.63377 (19)	0.0344 (10)	
N7	1.0188 (4)	0.7972 (3)	0.7672 (3)	0.078 (2)	
C1	0.8415 (5)	0.6056 (2)	0.7668 (3)	0.0549 (16)	
H1	0.904335	0.618576	0.775054	0.066*	
C2	0.8221 (5)	0.6006 (2)	0.6941 (3)	0.0524 (16)	
H2A	0.826355	0.630221	0.673115	0.063*	
H2B	0.758698	0.589500	0.684601	0.063*	
C3	0.8882 (5)	0.5689 (2)	0.6652 (4)	0.0658 (19)	
H3A	0.950780	0.581640	0.670649	0.079*	
H3B	0.871207	0.565745	0.617733	0.079*	
C4	0.8888 (5)	0.5227 (2)	0.6971 (3)	0.0572 (17)	
H4	0.824581	0.511206	0.690311	0.069*	
C5	0.9087 (6)	0.5288 (2)	0.7717 (4)	0.070 (2)	
H5A	0.904789	0.499462	0.793477	0.083*	
H5B	0.971870	0.540217	0.780910	0.083*	
C6	0.8412 (5)	0.5612 (2)	0.8002 (4)	0.0635 (19)	
H6A	0.778607	0.548362	0.794944	0.076*	
H6B	0.858017	0.565107	0.847498	0.076*	
C7	0.9501 (5)	0.4877 (2)	0.6668 (4)	0.073 (2)	
C8	1.0503 (6)	0.5018 (3)	0.6728 (7)	0.123 (4)	
H8A	1.088104	0.477493	0.657646	0.148*	
H8B	1.069297	0.508583	0.718555	0.148*	
H8C	1.057988	0.528361	0.645923	0.148*	
C9	0.9412 (6)	0.4425 (2)	0.7014 (4)	0.080 (2)	
H9A	0.976262	0.419861	0.679605	0.096*	
H9B	0.876463	0.433737	0.699288	0.096*	
H9C	0.965064	0.445159	0.747185	0.096*	
C10	0.9145 (6)	0.4823 (3)	0.5934 (4)	0.087 (3)	
H10A	0.847364	0.481055	0.589740	0.104*	
H10B	0.939042	0.454693	0.576106	0.104*	
H10C	0.934816	0.507779	0.568314	0.104*	
C11	0.4612 (4)	0.56539 (18)	0.8631 (3)	0.0423 (13)	
H11	0.411180	0.577990	0.836960	0.051*	
C12	0.4837 (4)	0.52141 (18)	0.8532 (3)	0.0441 (14)	
H12	0.448158	0.504071	0.821748	0.053*	
C13	0.5577 (4)	0.50251 (16)	0.8888 (3)	0.0420 (14)	
C14	0.6053 (4)	0.52938 (17)	0.9356 (3)	0.0424 (13)	
H14	0.656926	0.517832	0.961176	0.051*	
C15	0.5772 (4)	0.57269 (17)	0.9448 (3)	0.0374 (12)	
C16	0.6198 (4)	0.60330 (16)	0.9959 (3)	0.0367 (12)	
C17	0.6824 (4)	0.59046 (18)	1.0461 (3)	0.0449 (14)	
H17	0.703687	0.560529	1.048447	0.054*	
C18	0.7148 (4)	0.62130 (19)	1.0939 (3)	0.0451 (14)	
C19	0.6807 (4)	0.66469 (19)	1.0874 (3)	0.0463 (14)	
H19	0.699986	0.686794	1.118718	0.056*	
C20	0.6193 (4)	0.67539 (17)	1.0356 (3)	0.0427 (14)	
H20	0.597764	0.705229	1.031575	0.051*	
C21	0.5927 (5)	0.45537 (17)	0.8758 (3)	0.0467 (15)	
C22	0.5206 (6)	0.4271 (2)	0.8359 (4)	0.069 (2)	
H22A	0.546631	0.397907	0.826713	0.083*	
H22B	0.502358	0.442336	0.794572	0.083*	
H22C	0.466748	0.423211	0.860834	0.083*	
C23	0.6773 (5)	0.4610 (2)	0.8356 (3)	0.0541 (16)	
H23A	0.697107	0.431731	0.820942	0.065*	
H23B	0.727297	0.475013	0.862935	0.065*	
H23C	0.660902	0.479946	0.797502	0.065*	
C24	0.6195 (6)	0.43094 (19)	0.9396 (3)	0.066 (2)	
H24A	0.674710	0.444621	0.961201	0.079*	
H24B	0.631702	0.399531	0.930342	0.079*	
H24C	0.569221	0.432957	0.968352	0.079*	
C25	0.7818 (5)	0.6071 (2)	1.1505 (3)	0.0567 (17)	
C26	0.8676 (6)	0.5873 (4)	1.1234 (5)	0.120 (4)	
H26A	0.910772	0.577798	1.159708	0.144*	
H26B	0.896751	0.609995	1.097513	0.144*	
H26C	0.850336	0.561629	1.095629	0.144*	
C27	0.7395 (7)	0.5705 (3)	1.1907 (4)	0.092 (3)	
H27A	0.691614	0.583508	1.215619	0.110*	
H27B	0.787205	0.557289	1.220902	0.110*	
H27C	0.712667	0.547453	1.161280	0.110*	
C28	0.8094 (6)	0.6449 (3)	1.1956 (4)	0.080 (2)	
H28A	0.839265	0.668133	1.171246	0.096*	
H28B	0.852241	0.633980	1.231203	0.096*	
H28C	0.754921	0.657261	1.213779	0.096*	
C29	0.4201 (4)	0.79870 (16)	0.9050 (2)	0.0338 (11)	
H29	0.480377	0.795707	0.925430	0.041*	
C30	0.3486 (4)	0.80094 (17)	0.9433 (3)	0.0392 (13)	
H30	0.359940	0.798650	0.989402	0.047*	
C31	0.2590 (4)	0.80653 (18)	0.9161 (3)	0.0388 (12)	
C32	0.2475 (4)	0.80805 (17)	0.8481 (3)	0.0383 (12)	
H32	0.187760	0.811002	0.826861	0.046*	
C33	0.3234 (3)	0.80525 (14)	0.8110 (2)	0.0282 (11)	
C34	0.3181 (4)	0.80821 (15)	0.7388 (3)	0.0325 (11)	
C35	0.2392 (4)	0.81715 (17)	0.7002 (3)	0.0371 (12)	
H35	0.183134	0.821422	0.719666	0.044*	
C36	0.2411 (4)	0.82000 (18)	0.6322 (3)	0.0433 (13)	
C37	0.3238 (4)	0.81104 (18)	0.6071 (3)	0.0409 (13)	
H37	0.327773	0.810687	0.561089	0.049*	
C38	0.4007 (4)	0.80262 (16)	0.6483 (3)	0.0360 (12)	
H38	0.456772	0.796799	0.629517	0.043*	
C39	0.1785 (4)	0.8129 (2)	0.9579 (3)	0.0533 (16)	
C40	0.2047 (5)	0.8057 (3)	1.0309 (3)	0.0623 (19)	
H40A	0.150432	0.809804	1.055400	0.075*	
H40B	0.251911	0.827326	1.046096	0.075*	
H40C	0.228491	0.775386	1.037988	0.075*	
C41	0.1465 (6)	0.8617 (3)	0.9489 (4)	0.088 (3)	
H41A	0.122741	0.866425	0.903454	0.106*	
H41B	0.198465	0.881738	0.959562	0.106*	
H41C	0.098084	0.867868	0.978029	0.106*	
C42	0.1006 (5)	0.7811 (3)	0.9365 (3)	0.079 (3)	
H42A	0.053480	0.782398	0.967875	0.095*	
H42B	0.124441	0.750591	0.934742	0.095*	
H42C	0.073632	0.789765	0.893107	0.095*	
C43	0.1538 (5)	0.8337 (2)	0.5900 (3)	0.0556 (17)	
C44	0.0790 (5)	0.7983 (3)	0.5966 (3)	0.0637 (19)	
H44A	0.063246	0.797055	0.642089	0.076*	
H44B	0.101668	0.769130	0.583661	0.076*	
H44C	0.024327	0.806232	0.568209	0.076*	
C45	0.1215 (5)	0.8792 (3)	0.6118 (4)	0.077 (2)	
H45A	0.169995	0.901281	0.607323	0.092*	
H45B	0.107159	0.877614	0.657554	0.092*	
H45C	0.066445	0.887994	0.584432	0.092*	
C46	0.1737 (5)	0.8368 (3)	0.5172 (3)	0.074 (2)	
H46A	0.197704	0.808197	0.502977	0.089*	
H46B	0.219133	0.860290	0.511757	0.089*	
H46C	0.116924	0.843934	0.490634	0.089*	
C47	0.6375 (4)	0.68457 (16)	0.5348 (3)	0.0375 (13)	
H47	0.612814	0.713340	0.541770	0.045*	
C48	0.7019 (4)	0.68003 (18)	0.4902 (3)	0.0387 (13)	
H48	0.720955	0.705347	0.466922	0.046*	
C49	0.7394 (4)	0.63815 (17)	0.4789 (3)	0.0365 (12)	
C50	0.7071 (4)	0.60286 (17)	0.5134 (3)	0.0365 (12)	
H50	0.730446	0.573759	0.507019	0.044*	
C51	0.6411 (4)	0.60917 (15)	0.5575 (2)	0.0311 (11)	
C52	0.5974 (4)	0.57334 (16)	0.5923 (2)	0.0339 (12)	
C53	0.6129 (4)	0.52825 (16)	0.5825 (2)	0.0355 (12)	
H53	0.657859	0.519455	0.554159	0.043*	
C54	0.5644 (4)	0.49580 (17)	0.6130 (3)	0.0383 (13)	
C55	0.5055 (4)	0.51132 (17)	0.6586 (3)	0.0426 (14)	
H55	0.473641	0.490657	0.683642	0.051*	
C56	0.4935 (4)	0.55659 (17)	0.6673 (3)	0.0408 (13)	
H56	0.453087	0.566159	0.698505	0.049*	
C57	0.8098 (4)	0.6306 (2)	0.4276 (3)	0.0470 (14)	
C58	0.8408 (5)	0.6740 (2)	0.3985 (4)	0.070 (2)	
H58A	0.867141	0.693449	0.433517	0.085*	
H58B	0.788058	0.688909	0.375380	0.085*	
H58C	0.887117	0.667798	0.367739	0.085*	
C59	0.7637 (5)	0.6014 (3)	0.3725 (3)	0.066 (2)	
H59A	0.807705	0.595362	0.339930	0.079*	
H59B	0.710530	0.617215	0.351579	0.079*	
H59C	0.743732	0.573230	0.390812	0.079*	
C60	0.8915 (5)	0.6054 (3)	0.4608 (4)	0.068 (2)	
H60A	0.870490	0.576921	0.477827	0.081*	
H60B	0.919769	0.623297	0.496879	0.081*	
H60C	0.936594	0.599670	0.428924	0.081*	
C61	0.5756 (4)	0.44589 (17)	0.5985 (3)	0.0445 (14)	
C62	0.6150 (5)	0.43821 (18)	0.5342 (3)	0.0573 (18)	
H62A	0.575623	0.452384	0.499209	0.069*	
H62B	0.618411	0.406099	0.525802	0.069*	
H62C	0.676602	0.451148	0.535406	0.069*	
C63	0.4813 (5)	0.42335 (18)	0.5962 (3)	0.0517 (16)	
H63A	0.486641	0.392432	0.581507	0.062*	
H63B	0.438227	0.439499	0.565756	0.062*	
H63C	0.458854	0.423711	0.639969	0.062*	
C64	0.6387 (5)	0.42511 (19)	0.6548 (3)	0.0528 (16)	
H64A	0.608450	0.426894	0.695666	0.063*	
H64B	0.696750	0.441509	0.659562	0.063*	
H64C	0.650633	0.393805	0.644698	0.063*	
C65	0.9641 (5)	0.7705 (3)	0.7664 (3)	0.066 (2)	
C66	0.8937 (6)	0.7368 (4)	0.7677 (5)	0.104 (3)	
H66A	0.918449	0.710726	0.792002	0.125*	
H66B	0.873525	0.727973	0.722916	0.125*	
H66C	0.841527	0.748781	0.789234	0.125*	

### Atomic displacement parameters (Å^2^)

**Table:**

	*U*^11^	*U*^22^	*U*^33^	*U*^12^	*U*^13^	*U*^23^
V1	0.0403 (5)	0.0201 (4)	0.0304 (4)	0.0032 (3)	0.0057 (4)	−0.0015 (3)
V2	0.0537 (6)	0.0216 (4)	0.0288 (4)	−0.0004 (4)	0.0098 (4)	−0.0013 (3)
V3	0.0417 (5)	0.0184 (4)	0.0333 (5)	0.0000 (3)	0.0035 (4)	−0.0037 (3)
V4	0.0389 (5)	0.0166 (4)	0.0338 (5)	0.0008 (3)	0.0051 (4)	−0.0023 (3)
V5	0.0453 (5)	0.0181 (4)	0.0342 (5)	−0.0011 (4)	0.0106 (4)	−0.0014 (3)
V6	0.0525 (6)	0.0202 (4)	0.0278 (4)	−0.0028 (4)	0.0063 (4)	−0.0010 (3)
V7	0.0384 (5)	0.0176 (4)	0.0320 (4)	−0.0010 (3)	0.0045 (4)	−0.0013 (3)
O1	0.050 (2)	0.0265 (18)	0.051 (2)	0.0027 (16)	0.0064 (19)	−0.0082 (17)
O2	0.051 (2)	0.0229 (17)	0.0314 (18)	0.0007 (15)	0.0071 (16)	0.0010 (14)
O3	0.046 (2)	0.0170 (16)	0.0309 (18)	0.0007 (14)	0.0100 (15)	−0.0004 (13)
O4	0.052 (2)	0.0165 (16)	0.0301 (18)	−0.0028 (15)	0.0074 (16)	0.0017 (14)
O5	0.058 (3)	0.0305 (19)	0.038 (2)	−0.0041 (17)	0.0156 (18)	0.0035 (16)
O6	0.052 (2)	0.0200 (16)	0.0302 (18)	0.0016 (15)	0.0080 (16)	−0.0036 (14)
O7	0.050 (2)	0.0271 (18)	0.0283 (18)	−0.0083 (16)	0.0064 (16)	0.0025 (14)
O8	0.052 (2)	0.0240 (18)	0.048 (2)	0.0048 (16)	0.0019 (18)	−0.0043 (16)
O9	0.039 (2)	0.0199 (16)	0.0356 (18)	−0.0011 (14)	0.0034 (15)	−0.0073 (14)
O10	0.039 (2)	0.0163 (15)	0.0338 (18)	−0.0017 (14)	0.0080 (15)	−0.0005 (13)
O11	0.046 (2)	0.0186 (16)	0.043 (2)	−0.0003 (15)	0.0042 (17)	−0.0020 (15)
O12	0.040 (2)	0.0180 (16)	0.0391 (19)	−0.0022 (14)	0.0106 (16)	0.0000 (14)
O13	0.043 (2)	0.0163 (15)	0.0296 (17)	−0.0013 (14)	0.0064 (15)	−0.0036 (13)
O14	0.050 (2)	0.0242 (18)	0.048 (2)	0.0020 (16)	0.0129 (18)	−0.0005 (16)
O15	0.049 (2)	0.0196 (16)	0.0343 (19)	0.0002 (15)	0.0060 (16)	−0.0024 (14)
O16	0.056 (3)	0.034 (2)	0.039 (2)	0.0003 (18)	0.0073 (18)	−0.0029 (17)
O17	0.064 (3)	0.0278 (18)	0.038 (2)	−0.0145 (18)	0.0131 (19)	−0.0077 (16)
O18	0.043 (2)	0.031 (2)	0.087 (3)	−0.0017 (17)	−0.005 (2)	0.005 (2)
N1	0.053 (3)	0.024 (2)	0.029 (2)	−0.005 (2)	0.009 (2)	−0.0007 (18)
N2	0.057 (3)	0.025 (2)	0.030 (2)	0.000 (2)	0.006 (2)	−0.0010 (18)
N3	0.036 (2)	0.0137 (18)	0.039 (2)	0.0044 (16)	0.0042 (19)	−0.0056 (17)
N4	0.043 (3)	0.0144 (18)	0.033 (2)	−0.0004 (17)	0.0078 (19)	0.0004 (16)
N5	0.054 (3)	0.0144 (18)	0.030 (2)	−0.0052 (18)	0.009 (2)	−0.0013 (16)
N6	0.055 (3)	0.025 (2)	0.023 (2)	−0.0098 (19)	0.0014 (19)	−0.0020 (17)
N7	0.047 (4)	0.118 (6)	0.070 (4)	−0.009 (4)	0.009 (3)	0.003 (4)
C1	0.048 (4)	0.037 (3)	0.080 (5)	0.009 (3)	0.006 (3)	−0.006 (3)
C2	0.064 (4)	0.041 (3)	0.052 (4)	0.006 (3)	0.006 (3)	0.005 (3)
C3	0.076 (5)	0.062 (4)	0.061 (4)	0.011 (4)	0.015 (4)	−0.009 (4)
C4	0.062 (4)	0.048 (4)	0.062 (4)	0.006 (3)	0.007 (3)	−0.010 (3)
C5	0.078 (5)	0.047 (4)	0.081 (5)	0.021 (4)	−0.012 (4)	−0.007 (4)
C6	0.076 (5)	0.049 (4)	0.064 (4)	0.015 (3)	−0.005 (4)	0.008 (3)
C7	0.070 (5)	0.049 (4)	0.098 (6)	0.013 (4)	0.002 (4)	−0.021 (4)
C8	0.063 (6)	0.080 (6)	0.230 (14)	0.013 (5)	0.032 (7)	−0.053 (8)
C9	0.089 (6)	0.050 (4)	0.099 (6)	0.017 (4)	−0.007 (5)	−0.017 (4)
C10	0.103 (7)	0.074 (6)	0.086 (6)	0.016 (5)	0.030 (5)	−0.026 (5)
C11	0.058 (4)	0.033 (3)	0.037 (3)	−0.011 (3)	0.009 (3)	−0.003 (2)
C12	0.066 (4)	0.029 (3)	0.039 (3)	−0.014 (3)	0.009 (3)	0.000 (2)
C13	0.074 (4)	0.020 (2)	0.034 (3)	−0.009 (3)	0.017 (3)	−0.002 (2)
C14	0.061 (4)	0.024 (3)	0.044 (3)	−0.002 (2)	0.013 (3)	−0.005 (2)
C15	0.050 (3)	0.031 (3)	0.033 (3)	−0.001 (2)	0.011 (2)	−0.006 (2)
C16	0.052 (3)	0.017 (2)	0.042 (3)	0.001 (2)	0.010 (3)	−0.003 (2)
C17	0.055 (4)	0.028 (3)	0.052 (3)	0.004 (3)	0.004 (3)	−0.007 (3)
C18	0.053 (4)	0.040 (3)	0.042 (3)	0.004 (3)	0.005 (3)	−0.007 (3)
C19	0.062 (4)	0.035 (3)	0.042 (3)	−0.004 (3)	0.003 (3)	−0.009 (3)
C20	0.068 (4)	0.023 (3)	0.037 (3)	0.000 (3)	0.010 (3)	−0.008 (2)
C21	0.078 (4)	0.024 (3)	0.040 (3)	−0.006 (3)	0.019 (3)	−0.004 (2)
C22	0.103 (6)	0.026 (3)	0.078 (5)	−0.008 (3)	0.011 (4)	−0.014 (3)
C23	0.078 (5)	0.040 (3)	0.046 (3)	0.006 (3)	0.019 (3)	−0.004 (3)
C24	0.120 (7)	0.024 (3)	0.055 (4)	0.007 (3)	0.021 (4)	0.003 (3)
C25	0.052 (4)	0.052 (4)	0.064 (4)	0.003 (3)	−0.011 (3)	−0.001 (3)
C26	0.072 (6)	0.197 (12)	0.085 (6)	0.050 (7)	−0.028 (5)	−0.019 (7)
C27	0.117 (8)	0.070 (5)	0.080 (6)	−0.024 (5)	−0.044 (5)	0.018 (4)
C28	0.104 (7)	0.066 (5)	0.063 (5)	0.002 (4)	−0.037 (4)	0.001 (4)
C29	0.044 (3)	0.025 (2)	0.032 (3)	0.003 (2)	0.002 (2)	−0.002 (2)
C30	0.057 (4)	0.028 (3)	0.035 (3)	0.004 (2)	0.012 (3)	−0.009 (2)
C31	0.046 (3)	0.034 (3)	0.038 (3)	0.001 (2)	0.007 (2)	0.003 (2)
C32	0.033 (3)	0.032 (3)	0.050 (3)	0.002 (2)	0.004 (2)	−0.001 (2)
C33	0.037 (3)	0.014 (2)	0.035 (3)	0.0004 (19)	0.009 (2)	0.0013 (19)
C34	0.038 (3)	0.016 (2)	0.044 (3)	0.001 (2)	0.001 (2)	0.000 (2)
C35	0.041 (3)	0.029 (3)	0.041 (3)	0.000 (2)	0.001 (2)	0.003 (2)
C36	0.047 (3)	0.032 (3)	0.050 (3)	−0.004 (2)	−0.001 (3)	0.004 (3)
C37	0.055 (4)	0.032 (3)	0.036 (3)	−0.002 (2)	0.005 (3)	0.000 (2)
C38	0.044 (3)	0.023 (2)	0.041 (3)	−0.002 (2)	0.004 (2)	0.001 (2)
C39	0.050 (4)	0.068 (4)	0.044 (3)	0.008 (3)	0.015 (3)	0.004 (3)
C40	0.050 (4)	0.089 (5)	0.051 (4)	−0.001 (4)	0.018 (3)	−0.001 (4)
C41	0.091 (6)	0.090 (6)	0.089 (6)	0.047 (5)	0.047 (5)	0.021 (5)
C42	0.036 (4)	0.153 (8)	0.048 (4)	−0.011 (4)	0.002 (3)	0.014 (5)
C43	0.048 (4)	0.069 (4)	0.048 (4)	−0.003 (3)	−0.009 (3)	0.010 (3)
C44	0.053 (4)	0.089 (5)	0.048 (4)	−0.002 (4)	−0.006 (3)	−0.007 (4)
C45	0.067 (5)	0.068 (5)	0.090 (6)	0.018 (4)	−0.028 (4)	0.014 (4)
C46	0.061 (5)	0.103 (6)	0.056 (4)	−0.014 (4)	−0.013 (4)	0.029 (4)
C47	0.058 (4)	0.021 (2)	0.034 (3)	0.003 (2)	0.011 (3)	0.007 (2)
C48	0.053 (4)	0.032 (3)	0.031 (3)	0.001 (2)	0.003 (2)	0.003 (2)
C49	0.040 (3)	0.031 (3)	0.038 (3)	0.002 (2)	0.001 (2)	0.003 (2)
C50	0.042 (3)	0.024 (2)	0.043 (3)	0.004 (2)	0.002 (2)	−0.005 (2)
C51	0.048 (3)	0.021 (2)	0.023 (2)	−0.001 (2)	−0.002 (2)	−0.0010 (19)
C52	0.048 (3)	0.021 (2)	0.031 (3)	−0.004 (2)	−0.005 (2)	−0.001 (2)
C53	0.052 (3)	0.022 (2)	0.032 (3)	−0.001 (2)	−0.001 (2)	0.003 (2)
C54	0.052 (4)	0.025 (3)	0.035 (3)	−0.005 (2)	−0.009 (2)	0.000 (2)
C55	0.071 (4)	0.024 (3)	0.033 (3)	−0.011 (3)	0.003 (3)	0.002 (2)
C56	0.065 (4)	0.027 (3)	0.030 (3)	−0.012 (3)	0.006 (3)	0.004 (2)
C57	0.045 (3)	0.042 (3)	0.056 (4)	0.007 (3)	0.017 (3)	0.005 (3)
C58	0.070 (5)	0.053 (4)	0.095 (6)	0.005 (4)	0.042 (4)	0.014 (4)
C59	0.080 (5)	0.070 (5)	0.050 (4)	−0.002 (4)	0.028 (4)	−0.008 (3)
C60	0.050 (4)	0.080 (5)	0.074 (5)	0.022 (4)	0.013 (4)	0.009 (4)
C61	0.066 (4)	0.021 (3)	0.045 (3)	0.000 (3)	−0.006 (3)	−0.003 (2)
C62	0.101 (6)	0.021 (3)	0.051 (4)	−0.005 (3)	0.007 (4)	−0.009 (3)
C63	0.072 (4)	0.023 (3)	0.057 (4)	−0.009 (3)	−0.009 (3)	−0.002 (3)
C64	0.067 (4)	0.031 (3)	0.059 (4)	−0.001 (3)	−0.008 (3)	−0.002 (3)
C65	0.042 (4)	0.108 (6)	0.049 (4)	0.002 (4)	0.002 (3)	0.003 (4)
C66	0.068 (6)	0.115 (8)	0.128 (9)	−0.027 (5)	0.002 (6)	−0.009 (7)

### Geometric parameters (Å, º)

**Table:**

V1—O2	1.603 (3)	V4—N4	2.139 (4)
V1—O3	1.775 (3)	V4—N3	2.140 (4)
V1—O4	1.777 (3)	V4—O13	2.250 (3)
V1—O1	1.780 (4)	V5—O14	1.586 (4)
V2—O5	1.599 (4)	V5—O12	1.831 (3)
V2—O6	1.731 (3)	V5—O10	1.831 (3)
V2—O7	1.831 (4)	V5—O15	1.955 (4)
V2—N2	2.152 (5)	V5—O4	1.977 (3)
V2—N1	2.162 (4)	V5—V6	3.0362 (12)
V2—O3	2.332 (3)	V6—O16	1.614 (4)
V2—V3	3.0894 (12)	V6—O15	1.747 (3)
V3—O8	1.592 (4)	V6—O17	1.834 (4)
V3—O10	1.836 (3)	V6—N5	2.164 (4)
V3—O9	1.838 (3)	V6—N6	2.179 (4)
V3—O6	1.954 (4)	V6—O4	2.190 (4)
V3—O3	1.967 (3)	V7—O18	1.611 (4)
V4—O11	1.603 (3)	V7—O13	1.720 (3)
V4—O12	1.800 (3)	V7—O17	1.770 (4)
V4—O9	1.806 (4)	V7—O7	1.770 (3)
			
O2—V1—O3	106.21 (16)	C20—C19—H19	120.1
O2—V1—O4	105.91 (17)	C18—C19—H19	120.1
O3—V1—O4	113.62 (16)	N2—C20—C19	122.8 (5)
O2—V1—O1	106.06 (18)	N2—C20—H20	118.6
O3—V1—O1	112.90 (17)	C19—C20—H20	118.6
O4—V1—O1	111.45 (18)	C24—C21—C13	111.1 (5)
O5—V2—O6	105.34 (17)	C24—C21—C22	108.2 (5)
O5—V2—O7	100.55 (19)	C13—C21—C22	112.1 (6)
O6—V2—O7	104.80 (16)	C24—C21—C23	110.4 (6)
O5—V2—N2	90.13 (19)	C13—C21—C23	106.4 (4)
O6—V2—N2	88.73 (17)	C22—C21—C23	108.6 (5)
O7—V2—N2	159.66 (16)	C21—C22—H22A	109.5
O5—V2—N1	100.05 (17)	C21—C22—H22B	109.5
O6—V2—N1	148.79 (18)	H22A—C22—H22B	109.5
O7—V2—N1	87.80 (16)	C21—C22—H22C	109.5
N2—V2—N1	73.22 (17)	H22A—C22—H22C	109.5
O5—V2—O3	171.45 (18)	H22B—C22—H22C	109.5
O6—V2—O3	74.80 (14)	C21—C23—H23A	109.5
O7—V2—O3	87.57 (14)	C21—C23—H23B	109.5
N2—V2—O3	81.32 (15)	H23A—C23—H23B	109.5
N1—V2—O3	77.40 (14)	C21—C23—H23C	109.5
O5—V2—V3	140.43 (14)	H23A—C23—H23C	109.5
O6—V2—V3	35.35 (11)	H23B—C23—H23C	109.5
O7—V2—V3	96.50 (11)	C21—C24—H24A	109.5
N2—V2—V3	85.73 (12)	C21—C24—H24B	109.5
N1—V2—V3	116.12 (12)	H24A—C24—H24B	109.5
O3—V2—V3	39.52 (8)	C21—C24—H24C	109.5
O8—V3—O10	102.23 (18)	H24A—C24—H24C	109.5
O8—V3—O9	102.70 (17)	H24B—C24—H24C	109.5
O10—V3—O9	91.69 (14)	C28—C25—C18	112.7 (6)
O8—V3—O6	99.59 (18)	C28—C25—C27	108.1 (6)
O10—V3—O6	156.55 (16)	C18—C25—C27	110.4 (6)
O9—V3—O6	91.84 (14)	C28—C25—C26	109.1 (7)
O8—V3—O3	101.20 (16)	C18—C25—C26	109.1 (6)
O10—V3—O3	87.64 (13)	C27—C25—C26	107.3 (8)
O9—V3—O3	155.67 (16)	C25—C26—H26A	109.5
O6—V3—O3	79.73 (13)	C25—C26—H26B	109.5
O8—V3—V2	105.03 (14)	H26A—C26—H26B	109.5
O10—V3—V2	132.11 (10)	C25—C26—H26C	109.5
O9—V3—V2	119.07 (11)	H26A—C26—H26C	109.5
O6—V3—V2	30.83 (9)	H26B—C26—H26C	109.5
O3—V3—V2	48.98 (10)	C25—C27—H27A	109.5
O11—V4—O12	102.46 (17)	C25—C27—H27B	109.5
O11—V4—O9	103.13 (17)	H27A—C27—H27B	109.5
O12—V4—O9	100.16 (16)	C25—C27—H27C	109.5
O11—V4—N4	92.97 (17)	H27A—C27—H27C	109.5
O12—V4—N4	91.68 (16)	H27B—C27—H27C	109.5
O9—V4—N4	157.31 (15)	C25—C28—H28A	109.5
O11—V4—N3	94.14 (16)	C25—C28—H28B	109.5
O12—V4—N3	158.44 (16)	H28A—C28—H28B	109.5
O9—V4—N3	89.20 (16)	C25—C28—H28C	109.5
N4—V4—N3	73.61 (16)	H28A—C28—H28C	109.5
O11—V4—O13	174.98 (17)	H28B—C28—H28C	109.5
O12—V4—O13	79.69 (13)	C30—C29—N3	122.6 (5)
O9—V4—O13	80.81 (13)	C30—C29—H29	118.7
N4—V4—O13	82.39 (13)	N3—C29—H29	118.7
N3—V4—O13	82.74 (13)	C29—C30—C31	121.2 (5)
O14—V5—O12	101.91 (16)	C29—C30—H30	119.4
O14—V5—O10	103.27 (18)	C31—C30—H30	119.4
O12—V5—O10	93.35 (15)	C32—C31—C30	116.1 (5)
O14—V5—O15	100.93 (18)	C32—C31—C39	121.5 (5)
O12—V5—O15	92.85 (15)	C30—C31—C39	122.3 (5)
O10—V5—O15	153.17 (15)	C31—C32—C33	120.4 (5)
O14—V5—O4	101.27 (16)	C31—C32—H32	119.8
O12—V5—O4	156.45 (15)	C33—C32—H32	119.8
O10—V5—O4	85.33 (14)	N3—C33—C32	121.9 (5)
O15—V5—O4	78.71 (14)	N3—C33—C34	113.9 (4)
O14—V5—V6	105.74 (14)	C32—C33—C34	124.2 (5)
O12—V5—V6	122.11 (12)	N4—C34—C35	122.2 (5)
O10—V5—V6	126.91 (10)	N4—C34—C33	113.4 (4)
O15—V5—V6	32.71 (9)	C35—C34—C33	124.3 (5)
O4—V5—V6	46.05 (11)	C34—C35—C36	120.4 (5)
O16—V6—O15	104.53 (18)	C34—C35—H35	119.8
O16—V6—O17	101.61 (19)	C36—C35—H35	119.8
O15—V6—O17	105.18 (16)	C37—C36—C35	116.6 (5)
O16—V6—N5	90.95 (18)	C37—C36—C43	123.6 (5)
O15—V6—N5	87.92 (15)	C35—C36—C43	119.7 (5)
O17—V6—N5	158.69 (18)	C38—C37—C36	120.3 (5)
O16—V6—N6	93.69 (17)	C38—C37—H37	119.8
O15—V6—N6	153.46 (18)	C36—C37—H37	119.8
O17—V6—N6	89.49 (16)	N4—C38—C37	123.3 (5)
N5—V6—N6	72.41 (15)	N4—C38—H38	118.4
O16—V6—O4	172.24 (17)	C37—C38—H38	118.4
O15—V6—O4	77.70 (15)	C42—C39—C31	110.7 (5)
O17—V6—O4	84.77 (16)	C42—C39—C40	108.5 (6)
N5—V6—O4	81.66 (15)	C31—C39—C40	112.9 (5)
N6—V6—O4	81.87 (14)	C42—C39—C41	110.3 (7)
O16—V6—V5	141.52 (14)	C31—C39—C41	106.9 (5)
O15—V6—V5	37.21 (12)	C40—C39—C41	107.5 (6)
O17—V6—V5	95.34 (12)	C39—C40—H40A	109.5
N5—V6—V5	84.93 (11)	C39—C40—H40B	109.5
N6—V6—V5	120.97 (12)	H40A—C40—H40B	109.5
O4—V6—V5	40.53 (8)	C39—C40—H40C	109.5
O18—V7—O13	109.11 (18)	H40A—C40—H40C	109.5
O18—V7—O17	108.9 (2)	H40B—C40—H40C	109.5
O13—V7—O17	111.70 (17)	C39—C41—H41A	109.5
O18—V7—O7	108.6 (2)	C39—C41—H41B	109.5
O13—V7—O7	109.22 (16)	H41A—C41—H41B	109.5
O17—V7—O7	109.25 (17)	C39—C41—H41C	109.5
C1—O1—V1	129.3 (4)	H41A—C41—H41C	109.5
V1—O3—V3	113.55 (17)	H41B—C41—H41C	109.5
V1—O3—V2	148.84 (19)	C39—C42—H42A	109.5
V3—O3—V2	91.50 (12)	C39—C42—H42B	109.5
V1—O4—V5	115.67 (17)	H42A—C42—H42B	109.5
V1—O4—V6	140.14 (18)	C39—C42—H42C	109.5
V5—O4—V6	93.42 (14)	H42A—C42—H42C	109.5
V2—O6—V3	113.82 (17)	H42B—C42—H42C	109.5
V7—O7—V2	129.2 (2)	C45—C43—C44	110.6 (6)
V4—O9—V3	119.51 (18)	C45—C43—C36	109.7 (5)
V5—O10—V3	124.88 (19)	C44—C43—C36	109.3 (5)
V4—O12—V5	121.91 (18)	C45—C43—C46	108.5 (6)
V7—O13—V4	144.53 (19)	C44—C43—C46	108.4 (6)
V6—O15—V5	110.08 (18)	C36—C43—C46	110.4 (6)
V7—O17—V6	126.7 (2)	C43—C44—H44A	109.5
C15—N1—C11	117.6 (5)	C43—C44—H44B	109.5
C15—N1—V2	119.5 (3)	H44A—C44—H44B	109.5
C11—N1—V2	122.6 (4)	C43—C44—H44C	109.5
C20—N2—C16	118.8 (5)	H44A—C44—H44C	109.5
C20—N2—V2	121.5 (4)	H44B—C44—H44C	109.5
C16—N2—V2	119.5 (4)	C43—C45—H45A	109.5
C33—N3—C29	117.8 (4)	C43—C45—H45B	109.5
C33—N3—V4	119.5 (3)	H45A—C45—H45B	109.5
C29—N3—V4	122.6 (4)	C43—C45—H45C	109.5
C38—N4—C34	117.0 (5)	H45A—C45—H45C	109.5
C38—N4—V4	123.8 (4)	H45B—C45—H45C	109.5
C34—N4—V4	118.7 (3)	C43—C46—H46A	109.5
C47—N5—C51	118.4 (4)	C43—C46—H46B	109.5
C47—N5—V6	121.2 (3)	H46A—C46—H46B	109.5
C51—N5—V6	120.1 (3)	C43—C46—H46C	109.5
C56—N6—C52	117.3 (4)	H46A—C46—H46C	109.5
C56—N6—V6	123.2 (4)	H46B—C46—H46C	109.5
C52—N6—V6	119.0 (3)	N5—C47—C48	122.6 (5)
O1—C1—C6	110.9 (5)	N5—C47—H47	118.7
O1—C1—C2	111.6 (5)	C48—C47—H47	118.7
C6—C1—C2	110.9 (5)	C47—C48—C49	120.0 (5)
O1—C1—H1	107.7	C47—C48—H48	120.0
C6—C1—H1	107.7	C49—C48—H48	120.0
C2—C1—H1	107.7	C50—C49—C48	116.7 (5)
C1—C2—C3	112.1 (6)	C50—C49—C57	120.8 (5)
C1—C2—H2A	109.2	C48—C49—C57	122.4 (5)
C3—C2—H2A	109.2	C49—C50—C51	121.0 (5)
C1—C2—H2B	109.2	C49—C50—H50	119.5
C3—C2—H2B	109.2	C51—C50—H50	119.5
H2A—C2—H2B	107.9	N5—C51—C50	121.1 (4)
C2—C3—C4	112.5 (6)	N5—C51—C52	113.9 (4)
C2—C3—H3A	109.1	C50—C51—C52	124.8 (4)
C4—C3—H3A	109.1	N6—C52—C53	121.6 (5)
C2—C3—H3B	109.1	N6—C52—C51	114.1 (4)
C4—C3—H3B	109.1	C53—C52—C51	124.2 (5)
H3A—C3—H3B	107.8	C54—C53—C52	121.6 (5)
C3—C4—C7	115.2 (6)	C54—C53—H53	119.2
C3—C4—C5	108.1 (5)	C52—C53—H53	119.2
C7—C4—C5	114.2 (6)	C53—C54—C55	115.7 (5)
C3—C4—H4	106.2	C53—C54—C61	122.1 (5)
C7—C4—H4	106.2	C55—C54—C61	122.2 (5)
C5—C4—H4	106.2	C56—C55—C54	120.2 (5)
C6—C5—C4	112.1 (6)	C56—C55—H55	119.9
C6—C5—H5A	109.2	C54—C55—H55	119.9
C4—C5—H5A	109.2	N6—C56—C55	123.2 (5)
C6—C5—H5B	109.2	N6—C56—H56	118.4
C4—C5—H5B	109.2	C55—C56—H56	118.4
H5A—C5—H5B	107.9	C58—C57—C60	110.7 (6)
C1—C6—C5	111.3 (6)	C58—C57—C59	108.9 (6)
C1—C6—H6A	109.4	C60—C57—C59	109.1 (6)
C5—C6—H6A	109.4	C58—C57—C49	112.2 (5)
C1—C6—H6B	109.4	C60—C57—C49	108.0 (5)
C5—C6—H6B	109.4	C59—C57—C49	107.9 (5)
H6A—C6—H6B	108.0	C57—C58—H58A	109.5
C8—C7—C9	108.9 (7)	C57—C58—H58B	109.5
C8—C7—C4	111.5 (6)	H58A—C58—H58B	109.5
C9—C7—C4	109.8 (7)	C57—C58—H58C	109.5
C8—C7—C10	110.6 (8)	H58A—C58—H58C	109.5
C9—C7—C10	108.5 (6)	H58B—C58—H58C	109.5
C4—C7—C10	107.4 (6)	C57—C59—H59A	109.5
C7—C8—H8A	109.5	C57—C59—H59B	109.5
C7—C8—H8B	109.5	H59A—C59—H59B	109.5
H8A—C8—H8B	109.5	C57—C59—H59C	109.5
C7—C8—H8C	109.5	H59A—C59—H59C	109.5
H8A—C8—H8C	109.5	H59B—C59—H59C	109.5
H8B—C8—H8C	109.5	C57—C60—H60A	109.5
C7—C9—H9A	109.5	C57—C60—H60B	109.5
C7—C9—H9B	109.5	H60A—C60—H60B	109.5
H9A—C9—H9B	109.5	C57—C60—H60C	109.5
C7—C9—H9C	109.5	H60A—C60—H60C	109.5
H9A—C9—H9C	109.5	H60B—C60—H60C	109.5
H9B—C9—H9C	109.5	C62—C61—C63	108.2 (5)
C7—C10—H10A	109.5	C62—C61—C54	112.0 (5)
C7—C10—H10B	109.5	C63—C61—C54	109.0 (5)
H10A—C10—H10B	109.5	C62—C61—C64	110.3 (5)
C7—C10—H10C	109.5	C63—C61—C64	109.1 (5)
H10A—C10—H10C	109.5	C54—C61—C64	108.2 (4)
H10B—C10—H10C	109.5	C61—C62—H62A	109.5
N1—C11—C12	122.7 (6)	C61—C62—H62B	109.5
N1—C11—H11	118.6	H62A—C62—H62B	109.5
C12—C11—H11	118.6	C61—C62—H62C	109.5
C11—C12—C13	120.1 (5)	H62A—C62—H62C	109.5
C11—C12—H12	120.0	H62B—C62—H62C	109.5
C13—C12—H12	120.0	C61—C63—H63A	109.5
C12—C13—C14	117.1 (5)	C61—C63—H63B	109.5
C12—C13—C21	123.0 (5)	H63A—C63—H63B	109.5
C14—C13—C21	119.8 (6)	C61—C63—H63C	109.5
C15—C14—C13	120.0 (6)	H63A—C63—H63C	109.5
C15—C14—H14	120.0	H63B—C63—H63C	109.5
C13—C14—H14	120.0	C61—C64—H64A	109.5
N1—C15—C14	122.5 (5)	C61—C64—H64B	109.5
N1—C15—C16	113.3 (4)	H64A—C64—H64B	109.5
C14—C15—C16	124.2 (5)	C61—C64—H64C	109.5
N2—C16—C17	121.4 (5)	H64A—C64—H64C	109.5
N2—C16—C15	113.8 (5)	H64B—C64—H64C	109.5
C17—C16—C15	124.7 (5)	N7—C65—C66	178.0 (9)
C16—C17—C18	120.5 (5)	C65—C66—H66A	109.5
C16—C17—H17	119.8	C65—C66—H66B	109.5
C18—C17—H17	119.8	H66A—C66—H66B	109.5
C19—C18—C17	116.7 (6)	C65—C66—H66C	109.5
C19—C18—C25	122.6 (5)	H66A—C66—H66C	109.5
C17—C18—C25	120.7 (5)	H66B—C66—H66C	109.5
C20—C19—C18	119.9 (5)		
			
O2—V1—O1—C1	35.9 (5)	V2—N2—C16—C15	3.2 (6)
O3—V1—O1—C1	151.8 (5)	N1—C15—C16—N2	−8.3 (7)
O4—V1—O1—C1	−78.9 (5)	C14—C15—C16—N2	172.4 (5)
O2—V1—O3—V3	−174.21 (19)	N1—C15—C16—C17	169.0 (5)
O4—V1—O3—V3	−58.2 (2)	C14—C15—C16—C17	−10.3 (9)
O1—V1—O3—V3	70.0 (2)	N2—C16—C17—C18	0.6 (9)
O2—V1—O3—V2	−33.3 (4)	C15—C16—C17—C18	−176.5 (5)
O4—V1—O3—V2	82.7 (4)	C16—C17—C18—C19	−0.1 (9)
O1—V1—O3—V2	−149.1 (3)	C16—C17—C18—C25	178.3 (6)
O2—V1—O4—V5	169.68 (19)	C17—C18—C19—C20	−0.7 (9)
O3—V1—O4—V5	53.5 (2)	C25—C18—C19—C20	−179.1 (6)
O1—V1—O4—V5	−75.4 (2)	C16—N2—C20—C19	−0.6 (8)
O2—V1—O4—V6	36.9 (3)	V2—N2—C20—C19	173.3 (4)
O3—V1—O4—V6	−79.3 (3)	C18—C19—C20—N2	1.1 (9)
O1—V1—O4—V6	151.8 (3)	C12—C13—C21—C24	−139.4 (6)
O5—V2—O6—V3	174.2 (2)	C14—C13—C21—C24	45.0 (8)
O7—V2—O6—V3	−80.2 (2)	C12—C13—C21—C22	−18.3 (8)
N2—V2—O6—V3	84.4 (2)	C14—C13—C21—C22	166.2 (5)
N1—V2—O6—V3	30.9 (4)	C12—C13—C21—C23	100.3 (7)
O3—V2—O6—V3	3.06 (17)	C14—C13—C21—C23	−75.2 (6)
O18—V7—O7—V2	−125.5 (3)	C19—C18—C25—C28	−2.7 (10)
O13—V7—O7—V2	−6.6 (3)	C17—C18—C25—C28	179.0 (7)
O17—V7—O7—V2	115.8 (3)	C19—C18—C25—C27	118.3 (7)
O5—V2—O7—V7	137.4 (3)	C17—C18—C25—C27	−60.0 (8)
O6—V2—O7—V7	28.3 (3)	C19—C18—C25—C26	−124.0 (8)
N2—V2—O7—V7	−102.0 (5)	C17—C18—C25—C26	57.7 (9)
N1—V2—O7—V7	−122.7 (3)	C33—N3—C29—C30	0.7 (7)
O3—V2—O7—V7	−45.3 (3)	V4—N3—C29—C30	176.5 (4)
V3—V2—O7—V7	−6.7 (3)	N3—C29—C30—C31	−1.7 (8)
O11—V4—O9—V3	−169.1 (2)	C29—C30—C31—C32	2.2 (8)
O12—V4—O9—V3	−63.7 (2)	C29—C30—C31—C39	−175.0 (5)
N4—V4—O9—V3	56.7 (5)	C30—C31—C32—C33	−1.7 (7)
N3—V4—O9—V3	96.8 (2)	C39—C31—C32—C33	175.5 (5)
O13—V4—O9—V3	14.02 (19)	C29—N3—C33—C32	−0.2 (6)
O8—V3—O9—V4	166.5 (2)	V4—N3—C33—C32	−176.2 (3)
O10—V3—O9—V4	63.6 (2)	C29—N3—C33—C34	178.4 (4)
O6—V3—O9—V4	−93.2 (2)	V4—N3—C33—C34	2.4 (5)
O3—V3—O9—V4	−24.5 (5)	C31—C32—C33—N3	0.7 (7)
V2—V3—O9—V4	−78.1 (2)	C31—C32—C33—C34	−177.7 (5)
O14—V5—O10—V3	169.8 (2)	C38—N4—C34—C35	−4.0 (7)
O12—V5—O10—V3	66.7 (2)	V4—N4—C34—C35	168.7 (4)
O15—V5—O10—V3	−36.4 (4)	C38—N4—C34—C33	177.4 (4)
O4—V5—O10—V3	−89.7 (2)	V4—N4—C34—C33	−9.9 (5)
V6—V5—O10—V3	−68.5 (2)	N3—C33—C34—N4	4.8 (5)
O8—V3—O10—V5	−173.0 (2)	C32—C33—C34—N4	−176.7 (4)
O9—V3—O10—V5	−69.7 (2)	N3—C33—C34—C35	−173.8 (4)
O6—V3—O10—V5	28.9 (5)	C32—C33—C34—C35	4.8 (7)
O3—V3—O10—V5	86.0 (2)	N4—C34—C35—C36	0.6 (7)
V2—V3—O10—V5	63.3 (2)	C33—C34—C35—C36	179.0 (5)
O11—V4—O12—V5	166.0 (2)	C34—C35—C36—C37	3.4 (8)
O9—V4—O12—V5	60.0 (2)	C34—C35—C36—C43	−175.1 (5)
N4—V4—O12—V5	−100.5 (2)	C35—C36—C37—C38	−3.9 (8)
N3—V4—O12—V5	−54.5 (5)	C43—C36—C37—C38	174.6 (5)
O13—V4—O12—V5	−18.6 (2)	C34—N4—C38—C37	3.5 (7)
O14—V5—O12—V4	−161.7 (2)	V4—N4—C38—C37	−168.8 (4)
O10—V5—O12—V4	−57.3 (2)	C36—C37—C38—N4	0.5 (8)
O15—V5—O12—V4	96.5 (2)	C32—C31—C39—C42	52.8 (8)
O4—V5—O12—V4	28.7 (5)	C30—C31—C39—C42	−130.1 (6)
V6—V5—O12—V4	81.0 (2)	C32—C31—C39—C40	174.7 (6)
O18—V7—O13—V4	6.1 (4)	C30—C31—C39—C40	−8.3 (8)
O17—V7—O13—V4	126.6 (3)	C32—C31—C39—C41	−67.3 (8)
O7—V7—O13—V4	−112.4 (3)	C30—C31—C39—C41	109.7 (7)
O16—V6—O15—V5	−174.80 (18)	C37—C36—C43—C45	−120.7 (7)
O17—V6—O15—V5	78.6 (2)	C35—C36—C43—C45	57.7 (8)
N5—V6—O15—V5	−84.36 (19)	C37—C36—C43—C44	117.9 (6)
N6—V6—O15—V5	−42.9 (4)	C35—C36—C43—C44	−63.6 (7)
O4—V6—O15—V5	−2.44 (16)	C37—C36—C43—C46	−1.3 (9)
O18—V7—O17—V6	95.8 (3)	C35—C36—C43—C46	177.2 (6)
O13—V7—O17—V6	−24.8 (3)	C51—N5—C47—C48	−1.9 (8)
O7—V7—O17—V6	−145.7 (3)	V6—N5—C47—C48	−175.8 (4)
O16—V6—O17—V7	−116.2 (3)	N5—C47—C48—C49	0.0 (9)
O15—V6—O17—V7	−7.5 (3)	C47—C48—C49—C50	1.2 (8)
N5—V6—O17—V7	118.8 (4)	C47—C48—C49—C57	178.0 (5)
N6—V6—O17—V7	150.1 (3)	C48—C49—C50—C51	−0.5 (8)
O4—V6—O17—V7	68.2 (3)	C57—C49—C50—C51	−177.3 (5)
V5—V6—O17—V7	29.0 (3)	C47—N5—C51—C50	2.7 (7)
V1—O1—C1—C6	−74.3 (7)	V6—N5—C51—C50	176.6 (4)
V1—O1—C1—C2	50.0 (7)	C47—N5—C51—C52	−174.0 (5)
O1—C1—C2—C3	−179.8 (5)	V6—N5—C51—C52	−0.1 (6)
C6—C1—C2—C3	−55.5 (8)	C49—C50—C51—N5	−1.5 (8)
C1—C2—C3—C4	56.5 (8)	C49—C50—C51—C52	174.8 (5)
C2—C3—C4—C7	176.1 (6)	C56—N6—C52—C53	2.6 (7)
C2—C3—C4—C5	−54.8 (8)	V6—N6—C52—C53	−169.8 (4)
C3—C4—C5—C6	54.8 (8)	C56—N6—C52—C51	179.9 (5)
C7—C4—C5—C6	−175.6 (6)	V6—N6—C52—C51	7.5 (6)
O1—C1—C6—C5	−180.0 (6)	N5—C51—C52—N6	−4.7 (6)
C2—C1—C6—C5	55.4 (8)	C50—C51—C52—N6	178.7 (5)
C4—C5—C6—C1	−56.5 (9)	N5—C51—C52—C53	172.4 (5)
C3—C4—C7—C8	61.6 (10)	C50—C51—C52—C53	−4.1 (8)
C5—C4—C7—C8	−64.4 (10)	N6—C52—C53—C54	2.1 (8)
C3—C4—C7—C9	−177.6 (7)	C51—C52—C53—C54	−174.8 (5)
C5—C4—C7—C9	56.4 (9)	C52—C53—C54—C55	−5.7 (8)
C3—C4—C7—C10	−59.7 (9)	C52—C53—C54—C61	175.2 (5)
C5—C4—C7—C10	174.3 (7)	C53—C54—C55—C56	4.7 (8)
C15—N1—C11—C12	−0.4 (8)	C61—C54—C55—C56	−176.2 (5)
V2—N1—C11—C12	173.0 (4)	C52—N6—C56—C55	−3.6 (8)
N1—C11—C12—C13	−2.0 (8)	V6—N6—C56—C55	168.4 (4)
C11—C12—C13—C14	2.0 (8)	C54—C55—C56—N6	−0.1 (9)
C11—C12—C13—C21	−173.7 (5)	C50—C49—C57—C58	−176.3 (6)
C12—C13—C14—C15	0.4 (8)	C48—C49—C57—C58	7.1 (8)
C21—C13—C14—C15	176.2 (5)	C50—C49—C57—C60	−54.0 (7)
C11—N1—C15—C14	2.8 (8)	C48—C49—C57—C60	129.3 (6)
V2—N1—C15—C14	−170.8 (4)	C50—C49—C57—C59	63.8 (7)
C11—N1—C15—C16	−176.5 (4)	C48—C49—C57—C59	−112.8 (6)
V2—N1—C15—C16	9.9 (6)	C53—C54—C61—C62	−20.5 (8)
C13—C14—C15—N1	−2.8 (8)	C55—C54—C61—C62	160.4 (6)
C13—C14—C15—C16	176.4 (5)	C53—C54—C61—C63	−140.2 (5)
C20—N2—C16—C17	−0.2 (8)	C55—C54—C61—C63	40.7 (7)
V2—N2—C16—C17	−174.3 (4)	C53—C54—C61—C64	101.3 (6)
C20—N2—C16—C15	177.2 (5)	C55—C54—C61—C64	−77.8 (7)

### Hydrogen-bond geometry (Å, º)

**Table:**

*D*—H···*A*	*D*—H	H···*A*	*D*···*A*	*D*—H···*A*
C19—H19···O14^i^	0.95	2.55	3.417 (7)	152
C30—H30···O16^i^	0.95	2.38	3.122 (6)	135
C37—H37···O5^i^	0.95	2.43	3.264 (7)	146
C40—H40*B*···O16^i^	0.98	2.58	3.466 (8)	150
C48—H48···O8^i^	0.95	2.32	3.216 (6)	157

Symmetry code: (i) *x*, −*y*−1/2, *z*−1/2.
